# Purpose in life, loneliness and depression among patients with substance use disorders in the psychiatric hospitals in Ghana

**DOI:** 10.1371/journal.pone.0299391

**Published:** 2024-04-18

**Authors:** Anthony Kwabena Nkyi, Jerry Paul K. Ninnoni

**Affiliations:** 1 Department of Guidance and Counselling, Faculty of Educational Foundation, College of Education Studies, University of Cape Coast, Cape Coast, Ghana; 2 Department of Mental Health, School of Nursing and Midwifery, College of Health and Allied Sciences, University of Cape Coast, Cape Coast, Ghana; HEED Lab, LLC, UNITED STATES

## Abstract

Substance use disorder is a global mental health issue. Purpose in life, Loneliness and depression are key indicators of poor mental health, and people with substance use disorder are more likely to experience low purpose in life, depression, and loneliness. This study aims to further the understanding of purpose in life, depression and loneliness among substance use disorders (SUD) patients in three hospitals in Ghana. It uses a cross-sectional survey design. A total of 425 SUD inpatients were screened. Beck Depression Inventory-II, the revised UCLA Loneliness Scale, and the Purpose in Life Test were utilised to measure depression, loneliness, and purpose in life respectively. Data were analysed using the SPSS version 23 for Windows. Data from 378 participants were collected for this study using a cross sectional survey design; after data cleaning, frequency tables (for categorical variables) and descriptive statistics (for continuous variables) were used to summarise the demographics and the three outcome variables depression, purpose in life, and loneliness. Our findings indicate that overall, participants are more likely to experience low sense of purpose in life, depression, and loneliness compared to the general population. There was no statistically significant relationship between depression and loneliness (r = 0.030, p = 0.567). There was a statistically significant negative relationship between depression and purpose in life (r = -0.514, p < 0.001). There was a statistically significant positive relationship between purpose in life and loneliness (r = 0.147, p = 0.004). There was no gender difference in depression, purpose in life, and loneliness among SUDs patients in psychiatric hospitals. There were no significant differences in purpose in life, depression and loneliness based on marital status. It is anticipated that the findings of this study will inform policies and clinical practice regarding service provision for patients with SUDs.

## Background

Substance use Disorders (SUD) are characterized by escalated and sustained drug intake, loss of control over drug intake and craving (American Psychiatric Association, 2013). SUD occurs only in individuals whose drug-use opportunities develop into a maladaptive pattern of drug-taking and seeking [1]). Early substance use is associated with polysubstance as well as substance use disorders, cognitive difficulties, behavioural issues, interrupted education and social functioning, financial and legal problems, and morbidity and early mortality [2]. Problematic substance use also commonly co-occurs with mental ill-health, a broad term referring to mental illness and mental health problems. In the general Australian population, for example, the 2019 National Drug Strategy Household Survey (NDSHS) indicated that people with mental ill-health were twice as likely to smoke cigarettes daily, and 1.7 times as likely to have used any illicit substance in the past year compared to their peers without mental ill-health [3]. The [4] found students with a diagnosis of mental disorder were three to six times more likely to report using illicit drugs compared to those without mental ill-health [5]. Similar trends were also observed in other countries, such as the UK and the USA. (U.S. Department of Health and Human Services 2020); [6]. Studies focusing on substance use in young people with emerging mental ill-health are less common [7]. According to the World Health Organization (WHO) report in 2008, one of every five adults experienced mental disorders in the past year, and 29.2% had a history of mental illness during their lifetime [8]. Studies suggest a strong association between substance use and Mental Health Disorders. Assessment of patients in Kenya for co-occurring substance use disorders (SUD) and mental disorders (MD) indicate that substance abuse correlates significantly with mental health disorders [9, 10].

Depression is a mood disorder which is characterized by short-term emotional responses to a serious health condition associated with impaired daily functioning accompanied by symptoms, such as sadness and frustration, feelings of guilt, insensibility, and loss of interest [11]. Depression is a common psychiatric disorder affecting more than 300 million people worldwide (World Health Organization, 2017). Individuals suffering from depression tend to report less purpose in life [12] and more loneliness [13]. The extent to which severe psychological distress interferes with the relation between purpose and loneliness is not yet known. For example, purpose in life could serve as a psychological resource that protects against loneliness even while one is experiencing psychological distress. In contrast, it is also possible that severe psychological distress limits the benefits of purpose, and/or that purpose among individuals with depression is too low to provide protection. Loneliness is not static but is both malleable and tends to follow a normative trajectory across adulthood. Specifically, loneliness tends to be highest in young adulthood, decreases through middle and older adulthood, and then increases among the oldest old [14]. That is, despite the stereotype that older adulthood brings loneliness [15], feelings of loneliness tend not to increase until the very end of life, and, even then, do not match the levels of loneliness experienced in younger adulthood [14]. Despite its lower prevalence in middle and early older adulthood, loneliness remains a critical concern because it is a significant determinant of health. That is, as described above, individuals who feel lonely are at a greater risk of morbidity and premature mortality [16–18]. To the extent that loneliness is modifiable and can be treated [19], there is great promise that intervening to reduce loneliness will help improve health outcomes. There is, indeed, some empirical evidence that higher purpose in life is associated with lower loneliness. Among men over the age of 60, for example, feeling more purposeful was associated with less loneliness, independent of lifestyle and mental health factors [20]. Purpose and loneliness are likewise associated more broadly with midlife and older adults [21].

According to [22], depression is three to four times more prevalent among individuals diagnosed with SUD than among the general population. This reciprocal comorbidity has been observed for all classes of addictive drugs, including nicotine, alcohol, cannabis, opiates and psychostimulants [23].

Low purpose in life among patients with SUDs is consistent with decreased sense of purpose in life, with negative consequences of substance use [24, 25]. Also, loneliness and low purpose in life are associated with SUD among patients [24]. A low sense of meaning in life has been seen as both the cause and the effect of dependent drinking [26]. Alcohol use has been associated with a negative sense of purpose in life [26].

A recent study conducted in a psychiatric hospital in Ghana (Ankaful) reported a significant association between Purpose in Life and Loneliness [27]. However, the study was conducted in only one psychiatric hospital in Ghana. Furthermore, with a homogenous small sample size population, the external validity of the results might be limited, thus limiting the generalizability of the findings. This highlighted the need for further investigation. Thus, this study examined the relationship between purpose in life (PIL), loneliness and depression among SUD patients in all three psychiatric hospitals in Ghana.

### Research hypotheses

The research question that guided this study was: What is the level of purpose in life, loneliness and depression among SUDS patients in psychiatric hospitals in Ghana? There were three study hypotheses:

- H_01_: There is no significant relationship between depression, purpose in life, and loneliness, among SUDs patients in psychiatric hospitals.
- H_02_: There is no gender difference in depression, purpose in life, and loneliness, among SUDs patients in psychiatric hospitals.
- H_03_: There is no significant relationship between depression, purpose in life, loneliness, and marital status among SUDs patients in psychiatric hospitals.

## Methods

### Study design

We used a cross-sectional survey design.

### Population

The total patient populations of the three (3) psychiatric hospitals constituted the population of the study. At the time of study, the patient population for all three hospitals was approximately 577.

### Study setting

This study was conducted at the three psychiatric hospitals in Ghana: Ankaful Psychiatric Hospital in Cape Coast, Accra Psychiatric Hospital, and Pantang Hospital. Accra Psychiatric Hospital currently has 304 patients. The functions of the hospital include treatment, training, and drug rehabilitation in Accra and other parts of the country. The hospital is also a training facility for medical and nursing students.

Ankaful Psychiatric Hospital serves the Central, Western and Northern sectors of Ghana. The hospital currently has a total patient population of 137. It is responsible for treating, training, and rehabilitating mentally ill patients and providing other addiction services. It serves as a training centre for health training institutions in mental health across the country, including the University of Cape Coast Medical School. The hospital also receives psychiatric patients from all over Ghana and neighbouring countries, Benin, Burkina Faso, Ivory Coast, Nigeria, and Togo.

Pantang hospital is the largest psychiatric hospital (acreage-wise); the hospital currently has a patient population of about 136. The hospital is a training centre for nursing and medical students all over the country. Pantang hospital comprises twenty-eight departments, including six male and three female wards, an assessment unit and a drug rehabilitation centre. The hospital also operates a polyclinic, maternity, child welfare clinic and an eye clinic which serves the general population. The Out-Patients Department provides free psychiatric and Primary Health Care (PHC) services to over fifteen villages in its catchment area of about ten kilometers.

At the time of data collection, the total number of mental health nurses, medical practitioners and psychiatrists in the three hospitals was 693, 36 and 7 respectively.

### Sample and sampling procedure

The sample included 378 inpatients with SUDs in the three psychiatric hospitals in Ghana. The study population of the three (3) psychiatric hospitals of patients with substance use disorders constitute the total population. To sample participants, we used convenience and snowball sampling. Eligible participants were those who met: (1) primary diagnosis with SUDs according to the [28]; (2) spoke English; (3) were at least 18 years of age: (4) had had at least one month treatment in the hospital and, (5) had had contact with a clinician who was familiar with the patient’s functioning (to obtain clinician ratings of substance use disorders). The charts of the patients were screened by the project coordinator to determine the possible severity of the illness. Those with severe illness were excluded from the study.

A total of 577 inpatient adults were screened for eligibility. Of the 577 inpatients in the three hospitals, 152 were ineligible for study participation, primarily because they did not meet substance use criteria, had not been in residence for one month of treatment in the hospital, had had no contact with a clinician who was familiar with the patient’s functioning (to obtain clinician ratings of substance use disorders) and were severely ill patients who could not give informed consent. In addition, of the 425 participants who were eligible, 47 participants either did not complete the survey or declined to provide complete information necessary for data analyses. As a result, the final study sample consisted of 378 participants.

### Ethical consideration

The study was submitted to the Institutional Review Board (IRB) of the University of Cape Coast (UCC) before data collection. Upon approval, an ethical clearance was acquired from the IRB and an introductory letter from the researchers was submitted to the Administrators of the three hospitals. Additionally, participants were duly educated on the details and requirement of the study, the importance of their involvement, as well as the voluntary nature of the study. Formal consent was sought from those who willingly decided to engage in the study by signing a written informed consent form.

Anonymity and confidentiality of participants’ information were strictly observed. In this regard identity of participants were concealed. Neither names nor any identifiable information from participants were taken. Only the assigned index and numbers were used to identify the questionnaire during data entry. No aspect of information from participants was given out. The answered questionnaires were kept in a locked box and were only retrieved when needed for further entry or verification.

### Date collection procedures

The research investigators recruited and trained three field assistants with backgrounds in psychology and mental health nursing to collect data. The study was submitted to the Institutional Review Board (IRB) of the University of Cape Coast (UCC) before data collection. Upon approval, an ethical clearance was acquired from the IRB and an introductory letter from the researchers was submitted to the Administrators of the three hospitals.

The questionnaire consisted of four parts. The first part requested participants to complete personal demographic, and the other parts consisted of the purpose in life test (PIL), Beck Depression Inventory (BDI) and Revised University of California Los Angeles Loneliness Scale *(*R-UCLA) were used. Participants took about 30 to 40 minutes to complete the questionnaire. All the questionnaires were self-administered in English, with the two research investigators and care givers on hand at their wards from 26th February to 30th April 2022. The instruments were administered in English because all the participants spoke and read English. Approximately 9 weeks were used for the data collection because participants were contacted at different times. The research assistants explained the consent forms and items that needed further clarification, and administered the questionnaires to the participants The three research assistants also signed consent forms to indicate that whatever they did and undertook in the study would be confidential and that they would not disclose any information to anyone under any circumstances. Participants were informed that they could discontinue if they felt distressed. All participants signed a written informed consent form. Counsellors from the Counselling Centre of the University of Cape Coast were available to offer counselling to individuals. Covid-19 health and safety protocols, including social distancing and wearing of nose masks, were adhered to.

### Measures

The set included a socio-demographic questionnaire covering, among others, age, gender, education level, marital status, religion, and medication of participants, as well as questionnaires using Purpose in life (PIL), The Revised University of California Los Angeles Loneliness Scale (R-UCLA), and The Beck Depression Inventory II (BDI-II).

*Purpose in life (PIL)* was measured using the purpose in life test, a 20-item instrument rated on a 7-point scale, with a high score (6 to 7) indicative of clear meaning and purpose in life, an intermediate score (3 to 5) indicative of indecision, and a low score (1 to 2) indicative of a lack of clear meaning or purpose in life. The final score was calculated by adding the scores of each of the 20 statements to create a composite PIL score of between 20 and 140 points. Purpose in life scores greater than 112 indicate the presence of definite meaning and purpose in life; scores between 92 and 112 fall in the indecisive range; and scores less than 92 indicate the lack of a clear purpose and meaning in life [29]. The estimated Cronbach’s alpha for purpose in life is 0.94, and the Cronbach’s alpha in this current study is 0.89.

The *Revised University of California Los Angeles Loneliness Scale (R-UCLA)* was developed by [30] and is one of the most widely used instruments for measuring the subjective experience of loneliness. Participants were asked to respond to each item statement with responses of never, rarely, sometimes, or always. Higher scores on the loneliness scale indicate higher loneliness. The 20-item R-UCLA uses a four-point Likert scale ranging from “1 = never; 2 = rarely; 3 = sometimes; 4 = often” [30] and is a widely used, reliable and valid measurement of loneliness. Items that are asterisked (1, 5, 6, 9, 10, 15, 16, 19, 20) are reversed i.e., 1 = 4, 2 = 3, 3 = 2, 4 = 1. The total score will range from a low of 0 to a high of 60. The R-UCLA has a high internal consistency of 0.71, while Cronbach’s alpha in this current study is 0.72.

*The Beck Depression Inventory-II (BDI-II)* is a criterion for depression developed by [31]. The Beck Depression Inventory-II (BDI-II) is a widely used self-report questionnaire designed to assess the severity of depression symptoms and behavioral assessment in individuals. The BDI-II is scored by summing the ratings for the 21 items. Each item is rated on a 4-point scale ranging from 0 to 3. The maximum total score is 63. It has also been used in numerous treatment outcome studies and numerous studies with trauma-exposed individuals. The Cronbach’s alpha for depression is .92.

### 2. Data analysis

Data were imported into and analyzed using SPSS version 23 for Windows [32]. As having missing data affects Likert-type scales, replacement is suggested for the construction of scale scores [33]. Missing data from subjects with at least 90% of the Likert scale responses answered (i.e., number of missing survey items ≤ 2 for each survey instrument) were replaced with modes (the most frequent values) of the associated survey items, which allowed the minimization of the bias due to data imputation, and the retention of the sample size and, consequently, of statistical power in subsequent analyses [34–36]. Participants who did not answer 3 or more items for any survey instrument (i.e., BDI-II, R-UCLA, and PIL), or who did not reveal their gender or marital status, were excluded from the data analysis. After data cleaning, frequency tables (for categorical variables) and descriptive statistics (for continuous variables) were used to summarize the demographics and the three outcome variables, which are depression (measured as the total score of BDI-II), purpose in life (measured as the total score of PIL), and loneliness (measured as the total score of R-UCLA).

## 3. Results

Table 1 shows the demographics of the study subjects. The average age of the participants was 32.56 (SD = 10.87) years. Slightly over half of the participants were male (58.5%), while approximately 85.5% indicated that they had formal education. Almost half of the participants were currently single (68.8%). Majority of the participants were Christian (82.3%).

**Table 1:** Demographics: Participants at the three psychiatric hospitals in Ghana.

	N = 378	%
**Age (M(SD))**	**32.56 (10.87)**	
**Gender**		
Male	**221**	**58.5**
Female	**157**	**41.5**
**Education level**		
Secondary	**145**	**38.4**
Tertiary	**178**	**47.1**
**Missing response**	**55**	**14.5**
Marital status		
Married	**90**	**23.8**
Single	**260**	**68.8**
Divorced	**22**	**5.8**
Widowed	**6**	**1.6**
**Religion**		
Christian	**311**	**82.3**
Islam	**51**	**13.5**
Traditional	**9**	**2.4**
**Missing response**	**7**	**1.9**

Table 2: shows the descriptive statistics of depression (measured as the total score of BDI-II), purpose in life (measured as the total score of PIL), and loneliness (measured as the total score of R-UCLA). The mean scores were 16.74 (SD = 11.30), 99.61 (SD = 26.42), and 55.17 (SD = 6.95) for BDI-II, PIL, and R-UCLA, respectively, indicating that overall, participants of the study had low levels of depression, moderately high levels of purpose in life, and moderate levels of loneliness.

**Table 2:** Descriptive statistics of depression, purpose in life, and loneliness.

	Theoretical range	M	SD
Depression	0–63	16.74	11.30
Purpose in life	20–140	99.61	26.42
Loneliness	20–80	55.17	6.95

M = mean; SD = standard deviation.

To determine H_01_ (There is no significant relationship between depression, purpose in life, and loneliness, among SUDs patients in the psychiatric hospitals), three Pearson’s correlation coefficients [37] were performed.

For each analysis of the Pearson’s correlation coefficient, the following assumptions, including (1) the variables are continuous, i.e., interval or ratio variables; (2) the relationship between the variables needs to be linear; (3) there are no outliers in the data; and (4) the variables are normally distributed, were examined [37]. The first assumption was satisfied as the composite scores of three variables, depression (measured as the total score of BDI-II), purpose in life (measured as the total score of PIL), and loneliness (measured as the total score of R-UCLA), were ratio variables and continuous.

**Fig 1:**
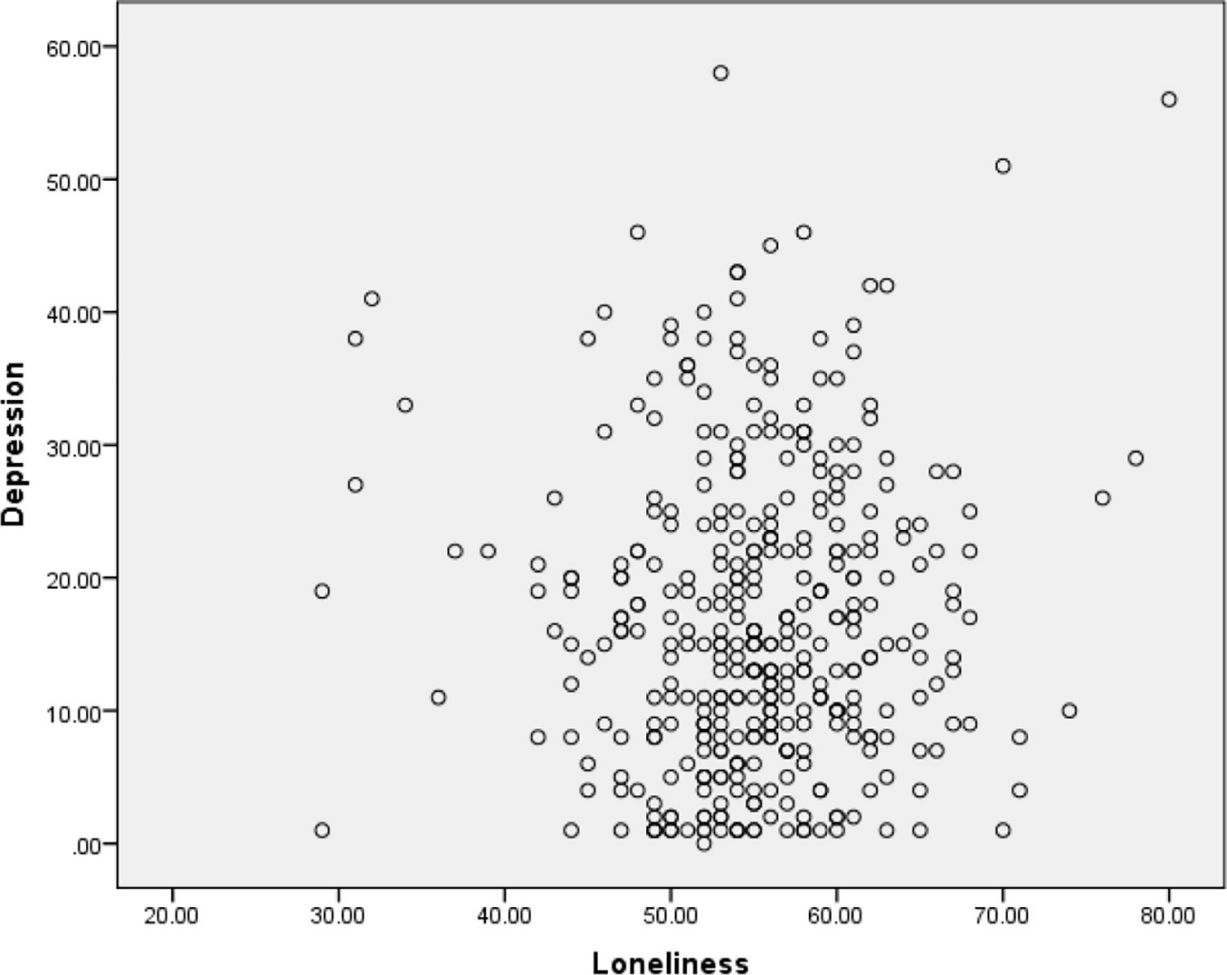
Scatter plot of depression and loneliness.

**Fig 2:**
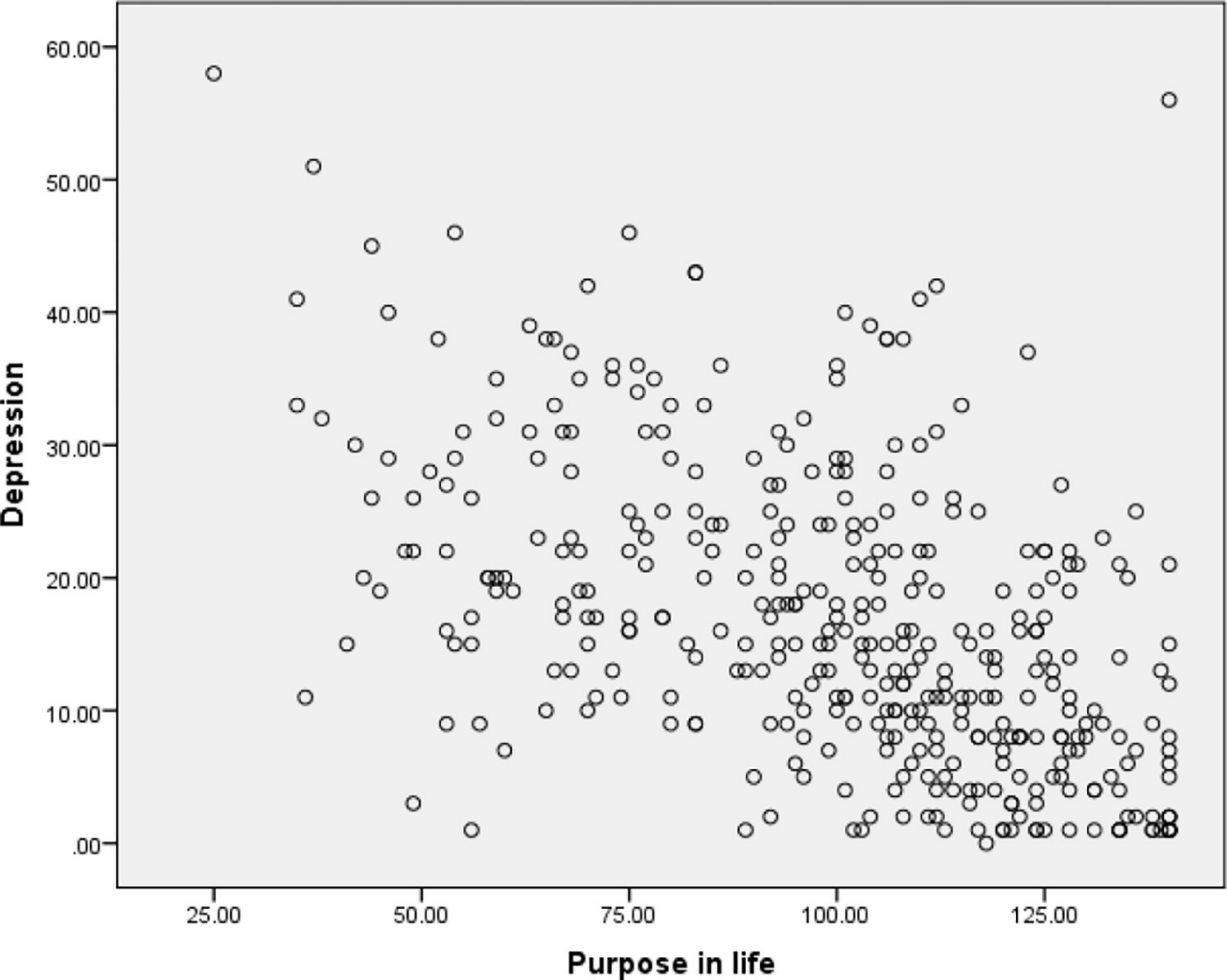
Scatter plot of depression and purpose in life.

**Fig 3:**
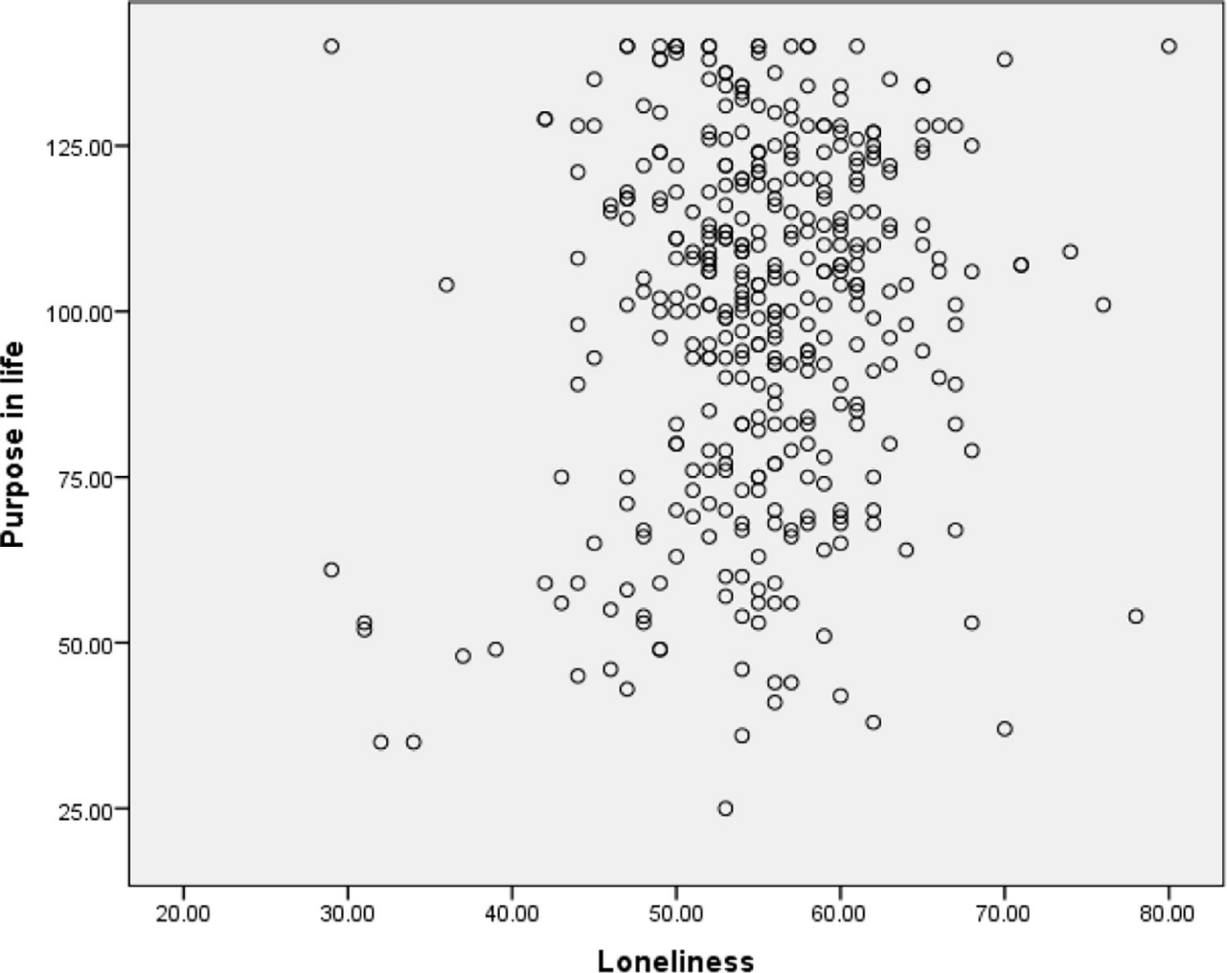
Scatter plot of purpose in life and loneliness.

The second assumption was checked using scatter plots to determine if the two variables (e.g., depression and purpose of life) exhibited a linear relationship. The scatter plots were also used to examine the third assumption, to ensure there were no significant outliers. The scatter plots are presented in Figs 1–3. It appeared that there was a linear relationship between the three variables and there were no outliers. Hence Assumptions 2 and 3 of Pearson’s correlation coefficients were satisfied.

The fourth assumption was checked using skewness and kurtosis. As suggested by [38, 39], an absolute skewness value ≤ 2 and an absolute kurtosis (excess) ≤ 4 for the data may be used as reference values for determining considerable normality. Since the values of skewness ranged from -0.52 to 0.73 and the values of kurtosis ranged from -0.51 to 2.19 (Table 3), it was concluded that the normality assumption was satisfied for Pearson’s correlation coefficients. As all assumptions were satisfied for Pearson’s correlation coefficients, it was appropriate to apply Pearson’s correlation coefficients to analyze the data.

**Table 3:** Pearson’s correlation coefficients.

Variable	Skewness	Kurtosis
Depression	0.73	0.23
Purpose in life	-0.52	-0.51
Loneliness	-0.33	2.19

The results indicate that there was no statistically significant relationship between depression and loneliness (r = 0.030, p = 0.567). There was a statistically significantly negative relationship between depression and purpose in life (r = -0.514, p < 0.001). This suggested that participants with lower levels of depression are purposeful and perceive life as more meaningful and, vice versa, participants who are purposeful and more meaningful would have lower levels of depression. Therefore, the more meaning in life one perceives, the less depressive symptoms they experience.

There was a statistically significantly positive relationship between purpose in life and loneliness (r = 0.147, p = 0.004). The results suggest that participants who are purposeful or perceive life as more meaningful would have higher levels of loneliness, and, vice versa, participants who had higher levels of loneliness would perceive lives as more meaningful and purposeful. (Table 4).

**Table 4:** Descriptive statistics of purpose in life, depression and loneliness.

Variables	r	*p*
Depression vs. loneliness	0.030	0.567
Depression vs. purpose in life	-0.514	< 0.001
Purpose in life vs. loneliness	0.147	0.004

r = Pearson’s correlation coefficient; *p* = *p-value*.

To determine H_02_ (There is no gender difference among depression, purpose in life, and loneliness, among SUDs patients in the psychiatric hospitals), three two-sample t-tests [37] were performed.

Two sample t-tests were used to determine if there was a difference in depression (measured as the total score of BDI-II), purpose in life (measured as the total score of PIL), and loneliness (measured as the total score of R-UCLA) between male and female patients.

The following three assumptions for two-sample t-tests were examined: a) independence of observations, b) the dependent variable should be approximately normally distributed, and c) there needs to be homogeneity of variances [37]. The independence assumption was satisfied as each patient was an independent individual and, hence, the observations were independent of one another.

The normality assumption was checked via skewness and kurtosis. As suggested by [38, 39], an absolute skewness value ≤ 2 and an absolute kurtosis (excess) ≤ 4 for the data may be used as reference values for determining considerable normality. Since the values of skewness ranged from -0.55 to 0.74 for males and from -0.53 to 0.72 for females, and the values of kurtosis ranged from -0.60 to 1.95 for males and from -0.40 to 2.61 for females (Table 5), it was concluded that the normality assumption was satisfied for the two-sample t-tests.

**Table 5:** Descriptive statistics (M (SD)) and results of ANOVAs of depression, purpose in life, and loneliness by marital status.

	Normality assumption				Levene’s test	
	Male		Female			
Variable	Skewness	Kurtosis	Skewness	Kurtosis	F	P
Depression	0.74	0.16	0.72	0.34	0.403	0.526
Purpose in life	-0.55	-0.60	-0.50	-0.40	0.260	0.610
Loneliness	-0.24	1.95	-0.53	2.61	1.814	0.179

F = F-statistic; p = p-value.

The assumption of homogeneity of variances was examined using Levene’s test. Since the p-values for Levene’s tests were all greater than 0.05 (Table 5), it was concluded that the assumption of homogeneity of variances for two-sample t-tests was satisfied. As all assumptions were satisfied for two-sample t-tests, it was appropriate to apply two-sample t-tests to analyze the data.

Mean (M) and standard deviation (SD) were reported, including sample size (n), t-statistic; (t), degrees of freedom(df) and p-value (p) as presented in Table 6.

**Table 6:** Assessment of the normality assumption for Pearson’s correlation coefficients.

	Male (N = 221)	Female (N = 157)	t	df	p
Depression	16.16 (11.10)	17.55 (11.57)	-1.184	376	0.237
Purpose in life	100.01 (25.93)	99.04 (27.18)	0.351	376	0.726
Loneliness	55.30 (7.29)	55.00 (6.45)	0.418	376	0.677

M = mean; SD = standard deviation; N = sample size; t = t-statistic; df = degrees of freedom; p = p-value.

The mean scores for depression were 16.16 (SD = 11.10) and 17.55 (SD = 11.57) for males and females, respectively. The results of the two-sample t-test indicated that there was no statistically significant difference in depression between males and females (t(376) = -1.184, p = 0.237).

The mean scores for purpose in life were 100.01 (SD = 25.93) and 99.04 (SD = 27.18) for males and females, respectively. The results of the two-sample t-test indicated that there was no statistically significant difference in purpose in life between males and females (t(376) = 0.351, p = 0.726).

**Fig 4:**
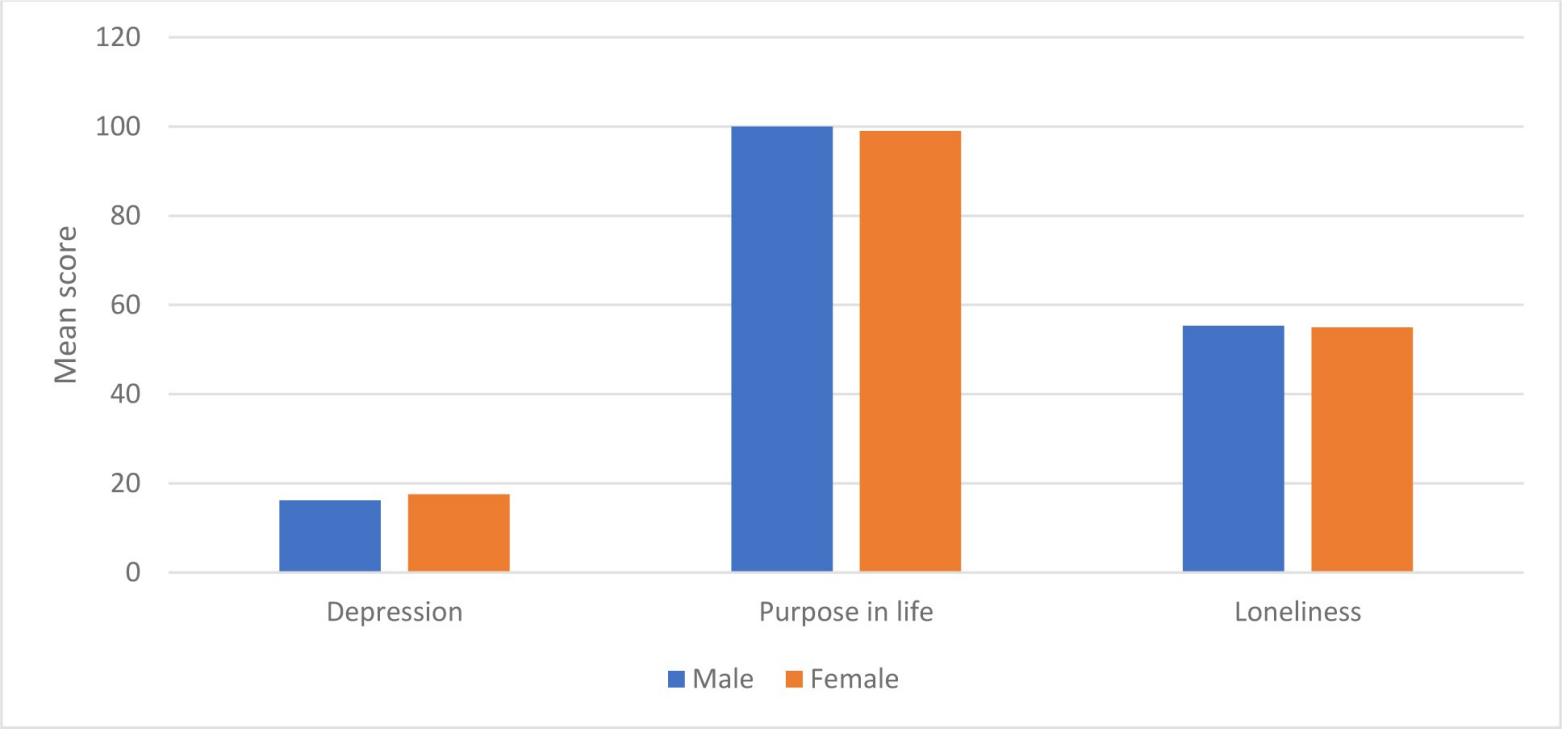
Mean scores of depression, purpose in life, and loneliness by gender.

The mean scores for loneliness were 55.30 (SD = 7.29) and 55.00 (SD = 6.45) for males and females, respectively. The results of the two-sample t-test indicated that there was no statistically significant difference in loneliness between males and females (t(376) = 0.418, p = 0.677). Fig 4 shows the mean scores of depression, purpose in life, and loneliness by gender.

To determine H_03_ (There is no significant relationship between depression, purpose in life, and loneliness, and marital status among SUDs patients in the psychiatric hospitals), three one-way analysis of variances (ANOVA) [40] were performed. One-way ANOVAs were performed to determine if there was a difference in depression (measured as the total score of BDI-II), purpose in life (measured as the total score of PIL), and loneliness (measured as the total score of R-UCLA) based on marital status. For any test, a p-value less than 0.05 indicated significance. All p-values were two-sided.

For ANOVAs, the following assumptions were checked: (a) independence of observations, (b) normality of the residuals, and (c) homoscedasticity (homogeneity of variances) [40, 41]. The assumption of independence of observations means that there was no relationship between the observations in each group or between the groups themselves. As participants of the study were independent individuals, the assumption of independence of observations was satisfied.

The assumption of normality of the residuals was checked using the skewness and kurtosis. As suggested by [38, 39], an absolute skewness value ≤ 2 and an absolute kurtosis (excess) ≤ 4 for the data may be used as reference values for determining considerable normality. Since the values of skewness ranged from -0.53 to 0.73 and the values of kurtosis ranged from -0.49 to 2.23 (Table 7), it was concluded that the normality assumption was satisfied for the ANOVAs.

**Table 7:** Assessment of the normality assumption and the homogeneity of variances assumption for two-sample t-tests.

	Levene’s test				Normality	
Variable	F	Df1	Df2	p	Skewness	Kurtosis
Depression	2.375	2	375	0.094	0.73	0.24
Purpose in life	0.829	2	375	0.437	-0.33	2.23
Loneliness	0.156	2	375	0.856	-0.53	-0.49

F = F-statistic; df1 = numerator degrees of freedom; df2 = denominator degrees of freedom; p = p-value.

The assumption of homoscedasticity (homogeneity of variances) was checked via Levene’s test [40]. Since the p-values for Levene’s tests were all greater than 0.05 (Table 7), it was concluded that the assumption of homogeneity of variances for ANOVAs was satisfied. As all assumptions were satisfied for ANOVAs, it was appropriate to apply ANOVAs to analyze the data. Note that marital status was regrouped into the following three categories: Never married, married, and single/separated/widows. Table 8 presents Descriptive statistics (M (SD)) and results of ANOVAs of depression, purpose in life, and loneliness by marital status.

**Table 8:** Assessment of the normality assumption and the homogeneity of variances assumption for ANOVAs.

	Never married	Married	Single/separated/widows	F	df1	df2	p
	(N = 83)	(N = 90)	(N = 205)				
Depression	16.35 (10.53)	16.30 (12.34)	17.09 (11.17)	0.214	2	375	0.808
Purpose in life	96.89 (25.98)	101.16 (26.86)	100.03 (26.45)	0.618	2	375	0.539
Loneliness	55.49 (6.59)	54.20 (7.03)	55.48 (7.05)	1.170	2	375	0.312

M = mean; SD = standard deviation; N = sample size; F = F-statistic; df1 = numerator degrees of freedom; df2 = denominator degrees of freedom; p = p-value.

The mean scores for depression were 16.35 (SD = 10.53), 16.30 (SD = 12.34), and 17.09 (SD = 11.07) for subjects who were never married, married, and single/separated/widows, respectively. There was no statistically significant difference in depression based on marital status (F(2, 375) = 0.214, p = 0.808).

The mean scores for purpose in life were 96.89 (SD = 25.98), 101.16 (SD = 26.86), and 100.03 (SD = 26.45) for subjects who were never married, married, and single/separated/widows, respectively. There was no statistically significant difference in purpose in life based on marital status (F(2, 375) = 0.618, p = 0.539).

**Fig 5:**
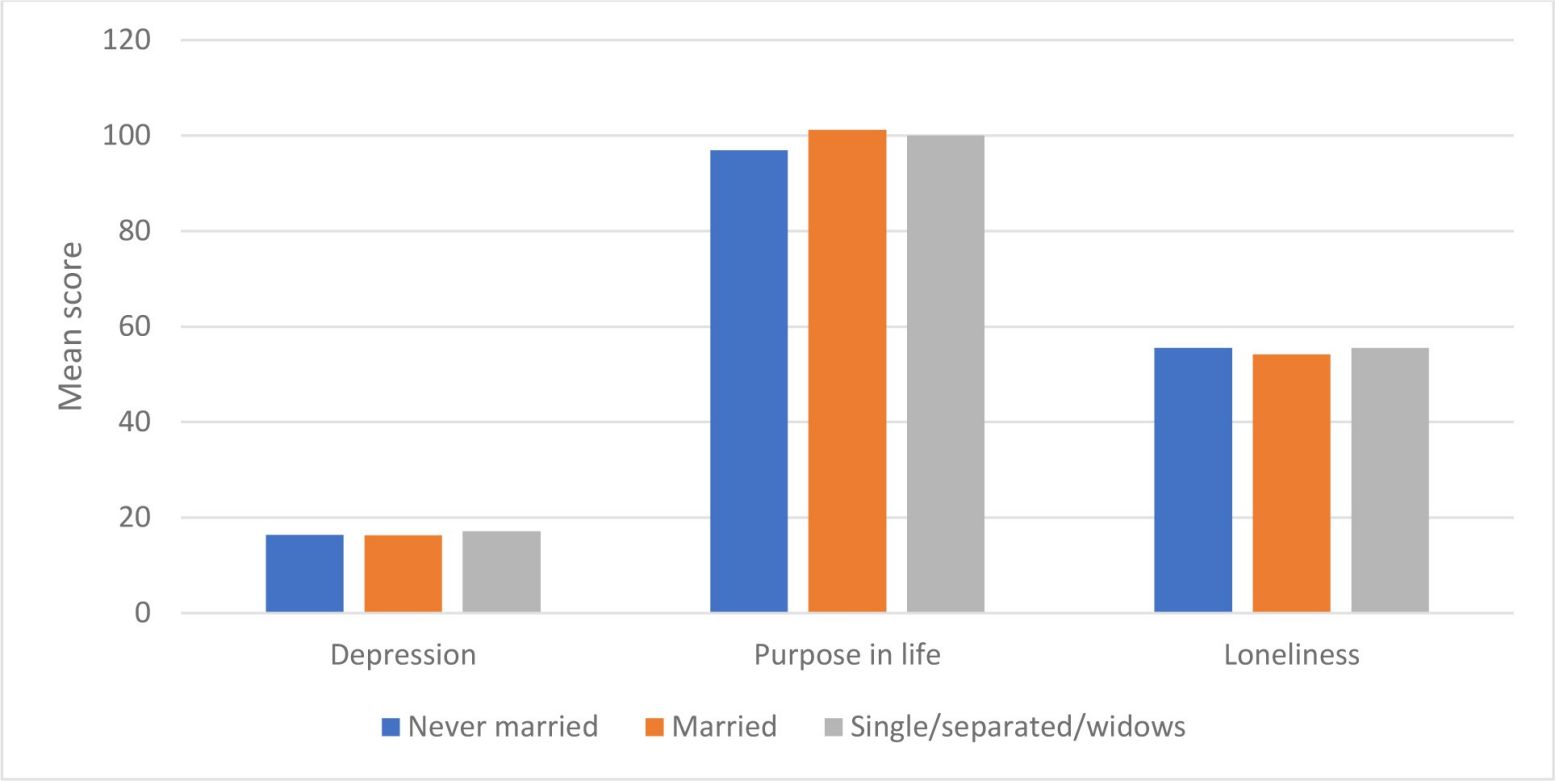
Mean scores of depression purpose in life, and loneliness by marital status.

The mean scores for loneliness were 55.49 (SD = 6.59), 54.20 (SD = 7.03), and 55.48 (SD = 7.05) for subjects who were never married, married, and single/separated/widows, respectively. There was no statistically significant difference in loneliness based on marital status (F(2, 375) = 1.170, p = 0.312). Fig 5 shows the mean scores of depression, purpose in life, and loneliness by marital status.

## Discussion

The present study explored correlations between purpose in life, loneliness, and depression among patients with substance use disorders in Ghana’s three psychiatric hospitals. The study identified that overall, participants had low levels of depression, moderately high levels of purpose in life, and moderate levels of loneliness. The low level of depression among SUD patients appears to be inconsistent with previous studies. [22], reported depression to be three to four times more prevalent among individuals diagnosed with SUD. The findings in this study might suggest that most patients are emotionally healthy and stable. This study was conducted among the clinical population, and one might expect a moderate or high level of depression, but this was not the case. The finding also suggests that being lonely does not necessarily suggest depression, and vice versa; however, there was some statistically significant relationship between purpose in life and depression. Depressed individuals may have reduced purpose in life, and, as one is depressed, meaning in life and the overall quality of life may reduce. There are many benefits of rehabilitation for SUD inpatients. The type of care can provide the structure and support they need to recover. It can also offer a sense of community and belonging for people who are feeling isolated. Thus, it may not be farfetched to suggest that participants have a sense of belonging. The evidence for cultural differences in loneliness is complex, and people from different societies and cultures may experience varying degrees of loneliness [42]. One study showed that living in a more collectivistic society was linked to greater loneliness. [43] and may, inevitably, be linked to depression.

These findings are consistent with previous research. [44] investigated the relationship between loneliness and depression and reported that loneliness is not an antecedent of depression. When depressed individuals engage in certain behaviours, it may lead to loneliness [44]. However, other studies found a significant relationship between loneliness and depression. A study by [45] reported a higher mean loneliness score among depressed patients than in the general population. This suggests that loneliness is a predictor of depression, contrary to the findings in our study.

The high level of PIL among SUD patients is in contrast to the results from a recent study in Ghana [27] and earlier studies [26, 46, 47]). This suggests that, generally, patients with SUDs in the survey have a high sense of meaning and are more purposeful in life. The current study revealed that SUD patients had a moderate level of loneliness which is inconsistent with the previous studies [27, 48]. However, other studies appear to be consistent with the results. [14] indicates that Loneliness is not static but is both malleable and tends to follow a normative trajectory across adulthood. Specifically, loneliness tends to be highest in young adulthood, decreases through middle and older adulthood, and then increases among the oldest old. The average age of the participants was 32.56 (SD = 10.87) years. These participants are in their young adulthood when loneliness appears to be high. Participants in the study might be experiencing emotional and social loneliness as defined by the absence of an attachment figure, or social isolation characterised by the lack of a social network [49]. Clinical and related statistics have demonstrated that loneliness is a common issue of modern people [50–52] pointed out that loneliness occurred when a person’s social network made him/her less satisfied than he/she expected. Some authors have reported that when social quality declines, the original network of relationships (loss or loss of loved ones, relocation) or lack of social skills (personality factors) could lead to strong loneliness [50, 53]. Hence, loneliness is a subjective feeling of unpleasant suffering caused by social defects [54], and long-term or severe loneliness may trigger certain emotional disorders and reduce mental health [51].

In the first hypothesis, we found no statistically significant relationship between depression and loneliness (r = 0.030, p = 0.567). However, studies have shown that loneliness is still significantly associated with depressive symptoms after controlling for demographic information (including gender, age, financial income, marital status) and risk factors that jointly influence loneliness and depression (including hostility, social support, stressors). Furthermore, there is a co-existence between depression and loneliness [55, 56]; people with depression are more likely to feel lonely due to actively avoiding people [57]. What is more, individuals with depression tend to lack confidence and have low self-evaluation, which may lead to feelings of loneliness [58]. In addition, social support is an important aspect to consider in influencing depression; that means loneliness, a perceived sense of lack of social support, lends support to this assertion.

Loneliness and depression are common health problems [59, 60], and appear to have similar characteristics, although their performance was different [61, 62]. We can conclude that loneliness, depression, and anxiety are inextricably linked [63, 64]. While others studies found a positive correlation between loneliness, anxiety, and depression [65–67] with loneliness significantly mediating anxiety and depression [68], our current study found no relationship between depression and loneliness.

There was a statistically significant negative relationship between depression and purpose in life (r = -0.514, p < 0.001). This suggests that participants with lower levels of depression would perceive lives as more meaningful and purposeful and, vice versa, subjects who perceived lives as more meaningful and purposeful would have lower levels of depression. This finding is consistent with previous studies [27, 69]. Meaning in life seemed to be inversely related to depression [69]. It may imply that patients with SUDs who are purposeful or perceive life as more meaningful will have lower levels of depression. The results of this study affirmed earlier findings that high purpose in life is a potent predictor of high well-being in patients with SUDs [70, 71].

Furthermore, there was a statistically significant positive relationship between purpose in life and loneliness (r = 0.147, p = 0.004). The results suggested that participants who are purposeful or perceive life as more meaningful would have higher levels of loneliness and, vice versa, participants who had higher levels of loneliness would perceive lives as purposeful and more meaningful. This finding is inconsistent with previous studies that linked a stronger sense of purpose in life to lower loneliness among older populations [20]. Since purpose in life and loneliness are correlated, it appears PIL is influenced by how much social support one views they have or don’t have [72]. It would be of interest to further study the relationship between purpose in life and loneliness to better understand why loneliness among participants is in the moderate level. It also appears that one can feel life has a purpose and is meaningful but lack relationships with people to share that meaning with, which could lead to loneliness and subsequent depressive symptoms. Studies have examined purpose in life as a psychological resource and a key component of well-being [73] that may help buffer against loneliness. Although little is known about the relationship between purpose and loneliness among SUDs patients, the findings show that purpose in life is not associated with lower levels of loneliness among patients with SUDS in psychiatric hospitals in Ghana.

Also, the results indicated no statistically significant difference in depression between males and females (t(376) = -1.184, p = 0.237). The results indicated no statistically significant difference in purpose in life between males and females (t(376) = 0.351, p = 0.726). Several studies reported contradictory findings on the relationship between the demographic variable, gender and depression [27, 74]. Whiles studies found no significant relationship between gender and depression [74], others reported a relationship between gender and depression [75]. Also, [27] observed that there was no difference between gender [76], while other studies suggest that gender predicts loneliness [77]. Other studies also reported men to be lonelier than women [77] and purpose in life was the only predictor of depression in participants.

In this study, there was no gender difference found in depression, purpose in life, and loneliness, among SUDs patients in psychiatric hospitals [78], found that women enjoyed higher levels of purpose in life, with the role of altruism accounting for much of the gender differences in purpose in life. Women were more likely to have altruistic behaviours and attitudes that have a strong effect in their life fulfilment of purpose. We, thus, suggest that men could also attain a similar level of purpose in life if social norms encouraged men to engage in altruistic behaviours as women do. Gender is one of the most frequently studied variables. The results of several studies showed that there are gender differences in depression, with women being significantly more likely to suffer from depression than men [79, 80]. For example, a cross-national epidemiological study covering the United States, Canada, France, Italy, New Zealand, Puerto Rico, West Germany, Lebanon, Taiwan, and South Korea showed that women were significantly more likely than men to suffer from depression [81].

Our final hypothesis was to investigate any significant relationship between depression, purpose in life, loneliness, and marital status. The results showed no statistically significant difference in purpose in life based on marital status. There was no statistically significant difference in depression and loneliness based on marital status. The results of the study are contrary to other studies. It has generally been reported that gender and marital status are associated with depression. A study found that married women showed more mental symptoms, and their positive mental health level and perceived health status were lower than those of men [82]. Furthermore, in most countries, people who were separated or divorced were markedly more likely to suffer from major depression than married people. Middle-aged and older men living alone showed more severe loneliness, depressive symptoms and suicidal ideation than women of the same age [83, 84], and widows would have a stronger sense of loneliness [85–87]. However, this may be due to the type of population that we studied. A person with SUD may have many challenges and, thus, may not be compared to other population of study.

### Implication for clinical practice

The results of the current study have implications for working effectively with individuals with substance use disorders. The present findings provide empirical evidence that purpose in life, loneliness and depression should not be overlooked in the life of patients with substance use disorders. It means that presence of purpose in life, depression and loneliness are real major concerns for mental health patients. Mental health professionals and health care providers should monitor whether patients with SUDs have meaning in life or feel depressed or lonely. Such patients might need clinical help. The study confirms that purpose in life is related to depression of SUD patients. Depression can occur as a result of loneliness and loneliness is highly associated with lack of social support. Comprehensive behavioral health services should be continuously adapted to address the multiple needs of this vulnerable population. For example, Substance Abuse Treatment Services and other behavioral therapies should be intensified through psychoeducational programs to ensure that individuals with substance use disorders receive behavioral health and social services. Health and Treatment providers should pay attention to how their patients experience meaning in their life or feel lonely or depressed. Consequently, psychotherapy should be encouraged in health facilities to enhance treatment outcomes and quality of life of patients.

### Limitations

The present study has several limitations. First, this study was a cross-sectional study, and its results could not provide a causal relationship. In terms of the study sample, all the participants in our study were patients with SUDS. Second, our findings were based on self-reported data, indicating the likelihood of reporting social desirability bias. which could have significant effect on the direction of data, given the perceived stigma associated with substance use disorder, depression and mental health issues in Ghana. Hence, further research may apply more professional surveys (e.g., Survey Monkey or Google Forums) to directly record time spent, eliminating coding data errors and controlling the answers at random.

Third, the assessment of depression symptoms was based on screening tools, and the results may differ if they were measured by clinical evaluation and diagnosis. In addition, some confounding variables may change over time (e.g., marital status).

## Conclusion

The purpose of this study was to investigate the relationships between purpose in life, depression and loneliness among substance use disorders (SUD) patients in three hospitals in Ghana. Based on the analysis conveyed, it can be concluded that overall participants had low levels of depression, moderately high levels of purpose in life, and moderate levels of loneliness. The findings from the study indicate (1) statistically significant negative relationship between depression and purpose in life; (2) statistically significant positive relationship between purpose in life and loneliness. However, there was: (1) no statistically significant relationship between depression and loneliness; (2) no gender difference in depression, purpose in life, and loneliness, among SUDs patients in psychiatric hospitals; and (3) no significant differences in purpose in life, depression and loneliness based on marital status. The findings of this study provide important insights into the relationship between purpose in life, depression and loneliness among substance use disorders and highlight the need for further research in this area.
